# First regulatory inspections measuring adherence to Good Pharmacy Practices in the public sector in Uganda: a cross-sectional comparison of performance between supervised and unsupervised facilities

**DOI:** 10.1186/s40545-016-0068-4

**Published:** 2016-05-04

**Authors:** Birna Trap, Kate Kikule, Catherine Vialle-Valentin, Richard Musoke, Grace Otto Lajul, Kim Hoppenworth, Dorthe Konradsen

**Affiliations:** Management Sciences for Health, Plot 15, Princess Anne Drive, Bugolobi, P.O. Box 71419, Kampala, Uganda; National Drug Authority, Plot 46 – 48 Lumumba Avenue, Kampala, Uganda; Harvard Pilgrim Health Care Institute, 133 Brookline Avenue, 6th Floor, Boston, MA 02215 USA; USAID/Securing Ugandans’ Right to Essential Medicines Program (SURE), Plot 15, Princess Anne Drive, Bugolobi, P.O. Box 71419, Kampala, Uganda; USAID/Uganda Health Supply Chain Program, Plot 15, Princess Anne Drive, Bugolobi, P.O. Box 71419 Kampala, Uganda

**Keywords:** Pharmacy practices, Pharmacy inspection, Pharmacy certification, Medicines management, Supervision, Pharmacy indicators, Public sector, Regulatory authority, Uganda

## Abstract

**Background:**

Since its inception, the Uganda National Drug Authority (NDA) has regularly inspected private sector pharmacies to monitor adherence to Good Pharmacy Practices (GPP). This study reports findings from the first public facility inspections following an intervention (SPARS: Supervision, Performance Assessment, and Recognition Strategy) to build GPP and medicines management capacity in the public sector.

**Methods:**

The study includes 455 public facilities: 417 facilities were inspected after at least four SPARS visits by trained managerial district staff (SPARS group), 38 before any exposure to SPARS. NDA inspectors measured 10 *critical*, 20 *major,* and 37 *minor* GPP indicators in every facility and only accredited facilities that passed all 10 critical and failed no more than 7 major indicators. Lack of compliance for a given indicator was defined as less than 75 % facilities passing that indicator. We assessed factors associated with certification using logistic regression analysis and compared number of failed indicators between the SPARS and comparative groups using two sample t-tests with equal or unequal variance.

**Results:**

57.4 % of inspected facilities obtained GPP certification: 57.1 % in the SPARS and 60.5 % in the comparative group (Adj. OR = 0.91, 95 % CI 0.45–1.85, *p* = 0.802). Overall, facilities failed an average of 10 indicators. SPARS facilities performed better than comparative facilities (9 (SD 6.1) vs. 13 (SD 7.7) failed indicators respectively; *p* = 0.017), and SPARS supported facilities scored better on indicators covered by SPARS. For all indicators but one minor, performance in the SPARS group was equal to or significantly better than in unsupervised facilities. Within the SPARS (intervention) group, certified facilities had < 75 % compliance on 7 indicators (all minor), and uncertified facilities on 19 (4 critical, 2 major, and 13 minor) indicators.

**Conclusions:**

Half of the Ugandan population obtains medicines from the public sector. Yet, we found only 3/5 of inspected public health facilities meet GPP standards. SPARS facilities tended to perform better than unsupervised facilities, substantiating the value of supporting supervision interventions in GPP areas that need strengthening. None compliant indicators can be improved through practices and behavioral changes; some require infrastructure investments. We conclude that regular NDA inspections of public sector pharmacies in conjunction with interventions to improve GPP adherence can revolutionize patient care in Uganda.

**Electronic supplementary material:**

The online version of this article (doi:10.1186/s40545-016-0068-4) contains supplementary material, which is available to authorized users.

## Background

Medicines are an essential and critical part of health care services in all cultures and societies. To ensure that patients receive adequate care, it is imperative to provide access to essential medicines and to trained health professionals who manage, prescribe, and dispense medicines appropriately.

In 1992, the first steps were taken to develop international standards for Good Pharmacy Practices (GPP) with the latest revision jointly published in 2011 by the International Pharmaceutical Federation and the World Health Organization [1, 2]. GPP is defined as the practice of pharmacy that provides optimal, evidence-based care for those who use pharmacists’ services [1, 2].

Several studies have found GPP implementation in the private sector to be suboptimal, but few have investigated how GPP is implemented in the public sector [3–7]. A study comparing GPP implementation between public and private pharmacies in Laos found that both sectors performed poorly, and no differences between sectors were observed in store management, labeling, or patient counseling. The authors recommended identifying interventions to improve pharmacy practices [8].

Since its inception in 1993, the National Drug Authority (NDA), an autonomous institution under Uganda’s Ministry of Health (MoH), has been responsible for ensuring that all medicines sold in Uganda are safe, effective, and handled in accordance to GPP standards. The legal framework is the same for the public and private sectors. It defines licensing and inspection, the practice and scope of pharmacy as well as supply chain integrity and medicines quality [9, 10].

In 2013, there were 604 licensed private sector pharmacies and 6140 licensed drug shops [11, 12]. Private facilities are regularly inspected by the NDA. Inspectors use an indicator-based tool that measures the key areas outlined in international GPP standards: suitability of premises, quality of dispensing, store management, and operating procedures. Until recently however, the NDA did not inspect public sector facilities on a regular basis. Instead, the MoH, district managers, and facility staff were responsible for GPP implementation in the 2867 government and 874 private not-for-profit (PNFP) facilities [11]. In Uganda, the public health sector is structured top-down. At district level, it is divided into District Hospitals (DH) and Health Centers (HC) IV, III, II, and I (i.e. Village Health Teams). Higher level facilities – hospitals and HC IV – have designated rooms for medicines storage and a trained stores manager often manages supplies. Lower level facilities – HCs II and III – often have only a cupboard for storage, and the nursing officer is responsible for all areas of management and use of medicines.

Close to half of Uganda’s population obtains their medicines from public sector facilities [13]. Yet, several constraints affect the quality of pharmacy services and patient welfare in Uganda’s public health care system: insufficient infrastructure, limited resources, poorly trained pharmaceutical staff especially at district level, and untrained medicines managers [11, 14, 15]. A 2012 national assessment of 3348 facility stores detailed severe deficiencies in infrastructure, pharmaceutical management tools, and storage facilities, including shelving. Given these challenges, requiring public sector facilities to meet the same practice and premises quality standards as the private sector has the potential to greatly improve patient care in Uganda.

In 2012, the MoH initiated a national Supervision, Performance Assessment, and Recognition Strategy (SPARS) to increase capacity in medicines management and pharmacy practices in the public sector, with the objective of building a foundation towards GPP certification of all public health care facilities [11, 16]. SPARS is based on supportive supervision. Supervisors are public district staff with formal training in medicines management including a two-week classroom instruction followed by 5 days of practical exercises. The role of supervisors is to visit participating public health care facilities about every 2 months. At each visit, supervisors use a standard instrument to measure the facility performance in five areas of medicines management: prescribing, dispensing, reporting and ordering, stores management, and stock management. The SPARS instrument includes a total of 25 indicators, 4-7 in each area. All indicators are scored 0/1. The data collected is entered on- or of-line into a data base that has inbuilt data checking and controls to clean and increase data quality [17]. Many of the SPARS indicators are similar or partly similar to the GPP inspection indicators with an indicator overlap of 73 %. During each visit, supervisors also provide tools such as stock cards, stock books, Uganda Clinical Guidelines, dispensing trays, dispensing envelopes, thermometers, and the national standard operating procedures manual. SPARS was initially rolled out in 45 districts (40 %) in Uganda, with encouraging results: after four SPARS visits, facilities achieved on average a 64 % improvement in medicines management and reached a score of 17.4 out of 25 [11, 16, 18].

Along with building medicines management knowledge through SPARS, Uganda initiated GPP inspections in the public sector to ensure equity of GPP implementation in the public and private sectors pharmacies. The aims of this study were a) to assess adherence to GPP at public health facilities in Uganda using results from the first public sector GPP inspections carried out in the country, and identify facility characteristics that influence GPP adherence and certification and b) to compare GPP certification and indicator scores between SPARS-supported facilities and unsupervised facilities.

## Methods

### Design and setting

The study is a cross-sectional, indicator-based comparison of adherence to national GPP requirements between SPARS-supported (intervention) and non-SPARS-supported (comparative) government and private not for profit health facility pharmacies in Uganda.

### Sampling

All 45 SPARS-supported districts and five randomly selected districts in the group of ten districts with no SPARS support participated in the study. In the SPARS-supported districts, facilities were considered ready for NDA inspection after receiving at least four SPARS supervisory visits. No other restrictions applied. The SPARS strategy is a national strategy implemented in 102 (91 %) districts by implementing partners with only ten districts not yet supported by SPARS limiting the comparative districts.

### Inspection tool

The tool in use for inspection of private pharmacies was modified for the public sector to increase clarity and reproducibility, and to address differences in staffing requirements in public facilities due to the shortage of pharmacists. Revisions were based on international GPP standards, the WHO rational drug use indicators, and SPARS indicators [1, 2, 5, 18, 19]. The revised tool was finalized in April 2013 after being piloted in eight inspections across two districts. The final tool applies both retrospective and prospective data collection through direct observations, record reviews, and questions. It includes 79 GPP indicators. Twelve indicators record general administrative information about the pharmacy, its ownership and staffing. The remaining 67 indicators are classified as critical (10), major (20), and minor (37). They assess the premises (9 critical, 8 major, 12 minor), dispensing quality (1 critical, 4 major, 14 minor), stores management (7 major, 8 minor), and operating requirements (1 major, 3 minor). 47 indicators assess performance in either the store or the dispensary, and 30 (45 %) assess performance at both locations. The list of all assessed indicators, their classification, and overlap with SPARS indicators are presented in Additional file 1.

Similarities between the GPP inspection and SPARS tools are inherent since SPARS aims to strengthen GPP implementation. 49 (73 %) GPP indicators are similar to SPARS indicators with 40 identical and 9 very similar indicators. The overlap is highest in the areas of stores management (93 %), premises (72 %), dispensing requirements (63 %) and operating requirements (50 %). Highest overlap of identical indicators is for major indicators (18/20) (90 %), followed by critical indicators (8/10) (80 %), and minor indicators with only (23/37) 62 % overlap.

### Certification

To earn GPP certification, facilities must comply with all critical indicators and not fail more than 7 major indicators. If certified, the number and type of failed indicators, if any, will determine the comments provided: no comments, minor comments, or major comments. Certification with minor comments indicates that the facility failed only minor indicators.

### Data collection

Intervention facilities were inspected between May 2013 and March 2014 and comparative facilities between January to March 2014. Inspections were planned in rounds of eight inspections each covering a five-day period. The NDA scheduled inspections according to inspectors’ availability and planned district inspection rounds. Forty-three trained and experienced national drug inspectors undertook the inspections. In preparation, they received a one-day training session on using the inspection tool and entering data electronically. They were not informed if they inspected an intervention or comparative district. They could not inspect their own residential district. After data entry, inspection results were uploaded to a central database and formed the basis of automatically-generated inspection reports. Eight inspectors surveyed both the comparative and intervention facilities. On average, they inspected 10 (range 1 to 37) intervention facilities and five (range 1 to 8) comparative facilities.

### Data analysis

Health facility characteristics were compared between facilities exposed to SPARS and those with no SPARS exposure using the Pearson chi-square tests together with Fisher’s exact tests. The proportions of facilities earning certification by characteristics were calculated. The association between certification status and health facility characteristics was assessed using bivariate logistic regression analysis to calculate odds ratios (ORs) and 95 % confidence intervals (CIs). Multivariable logistic regression, considering characteristics that were significant in the bivariate analysis, was used to estimate adjusted ORs (Adj. ORs) and CIs. The proportion of facilities passing each indicator was calculated and comparisons made by health facility exposure to SPARS and comparison group not exposed to SPARS. The “compliance score” was defined for each indicator as the percentage of facilities receiving a ‘pass’, i.e., the percentage of compliant facilities for that indicator. The Pearson chi-square test together with Fisher’s exact test were further used to assess the relationship between exposure to SPARS and the compliance score. To determine if a relationship existed, Simple Sequentially Rejective Multiple Test Procedure of adjusting the critical level was used to account for multiple testing of all the indicators [20]. The average number of indicators failed was calculated and compared by exposure to SPARS. Two sample t-tests with equal or unequal variances were used to compare if the difference of average number of failed indicators between the SPARS exposed and non-SPARS exposed facilities across the four certification categories (certified without comments, certified with minor or major comments, not certified) were significant. All statistical analyses were done using STATA software version13.1.

### Ethical considerations

The study is a retrospective analysis of data collected under NDA legal requirement to implement GPP and does not involve human subjects or data.

## Results

Inspections were carried out in 493 facilities. A total of 38 inspections were excluded from the analysis as one or more critical indicators were not recorded, leaving a total of 455 inspections eligible for analysis of which 417 were in intervention and 38 in comparative facilities. The intervention (SPARS-supported) inspected facilities were comparable to the comparative facilities with regards to ownership, and level of care. There were regional differences between the intervention and comparative facilities with 60 % of the comparative facilities being the in central region as compared to 32 % of the intervention facilities in the same region (*p* < 0.001). Comparative districts only represent three of the four regions excluding the Eastern Region. See Table 1.

**Table 1:** Comparison of health facility characteristics of comparative and intervention groups

	Reference	Intervention	Total	*p*-value
	# (%)	# (%)	# (%)	
Number of facilities (#)/(%)	38 (8.4)	417 (91.6)	455 (100)	
Ownership				
Government	30 (78.9)	357 (85.6)	387 (85.1)	0.270
PNFP	8 (21.1)	60 (14.4)	68 (14.9)	.
Level of care				
Health Center II	16 (42.1)	217 (52.0)	233 (51.2)	0.258
Health Center III	14 (36.9)	149 (35.7)	163 (35.8)	
Health Center IV	4 (10.5)	32 (7.7)	36 (7.9)	
Hospital	4 (10.5)	19 (4.6)	23 (5.1)	
Regions				
Central	23 (60.5)	121 (29.0)	144 (31.6)	<0.001
Northern	8 (21.1)	101 (24.2)	109 (24.0)	
Western	7 (18.4)	102 (24.5)	109 (24.0)	
Eastern	0 (0.0)	93 (22.3)	93 (20.4)	

### GPP certification and reasons for failure

Overall, the proportion of public health facilities which obtained accreditation was 57.4 % (Table 2). The percentage of certified SPARS-supported facilities and comparative facilities was 57.1 and 60.5 % respectively, this difference was not significant (Adj. OR = 0.91, 95 % CI 0.45–1.85, *p* = 0.802). The proportion of certified PNFP facilities (58.3 %) was slightly higher than that of government facilities (57.1 %) and the difference was not significant (Unadj. OR = 1.07, 95 % CI 0.64–1.81, *p* = 0.792). We found that facilities in the Northern Region had significantly higher odds of certification compared to those in the Central region (Adj. OR = 2.66, 95 % CI 1.54–4.61, *p* < 0.001). The odds of certification of facilities in the Western and Eastern regions were not significantly higher than those in the Central region (Adj. OR = 1.16, 95 % CI 0.70–1.92 and Adj. OR = 0.67, 95 % CI 0.39–1.14 respectively).

**Table 2:** Certification status and confidence interval by arm, ownership, level of care and regions for all facilities

No. certified/n (%)		Unadj. OR (95 % CI)	Adj. OR (95 % CI)
Total	261/455 (57.4)		
Arm			
Comparative	23/38 (60.5)	1.00	1.00
Intervention	238/417 (57.1)	0.87 (0.44–1.71)	0.91 (0.45–1.85)
Ownership			
Government	203/387 (57.1)	1.00	
PNFP	35/68 (58.3)	1.07 (0.64–1.81)	
Level of care			
Health Center II	125/233 (53.6)	1.00	
Health Center III	98/163 (60.1)	1.30 (0.87–1.95)	
Health Center IV	20/36 (55.6)	1.08 (0.53–2.19)	
Hospital	18/23 (78.3)	3.11 (1.12–8.66)*	
Region			
Central	77/144 (53.5)	1.00	1.00
Northern	82/109 (75.2)	2.64 (1.53–4.55)**	2.66 (1.54–4.61)**
Western	62/109 (56.9)	1.14 (0.70–1.89)	1.16 (0.70–1.92)
Eastern	40/93 (43.0)	0.66 (0.39–1.11)	0.67 (0.39–1.14)

* *p* < 0.05, ** *p* < 0.001

Within the group of SPARS-supported facilities, slightly more PNFP facilities were certified (58.3 %) than government facilities (56.9 %), but the difference was not significant (OR = 1.06, 95 % CI 0.61–1.85; *p* = 0.831). Certification was significantly more frequent for hospitals (89.5 %; Adj. OR = 8.92 95 % CI 1.97–40.51, *p* = 0.005) compared to HC IIs (52.5 %). Similar regional differences in the proportion of certified facilities were observed in this group; with facilities in the Northern region having significantly higher odds of certification compared to those in the Central region (Adj. OR = 2.75, 95 % CI 1.53–4.93, *p* = 0.001).

**Fig. 1:**
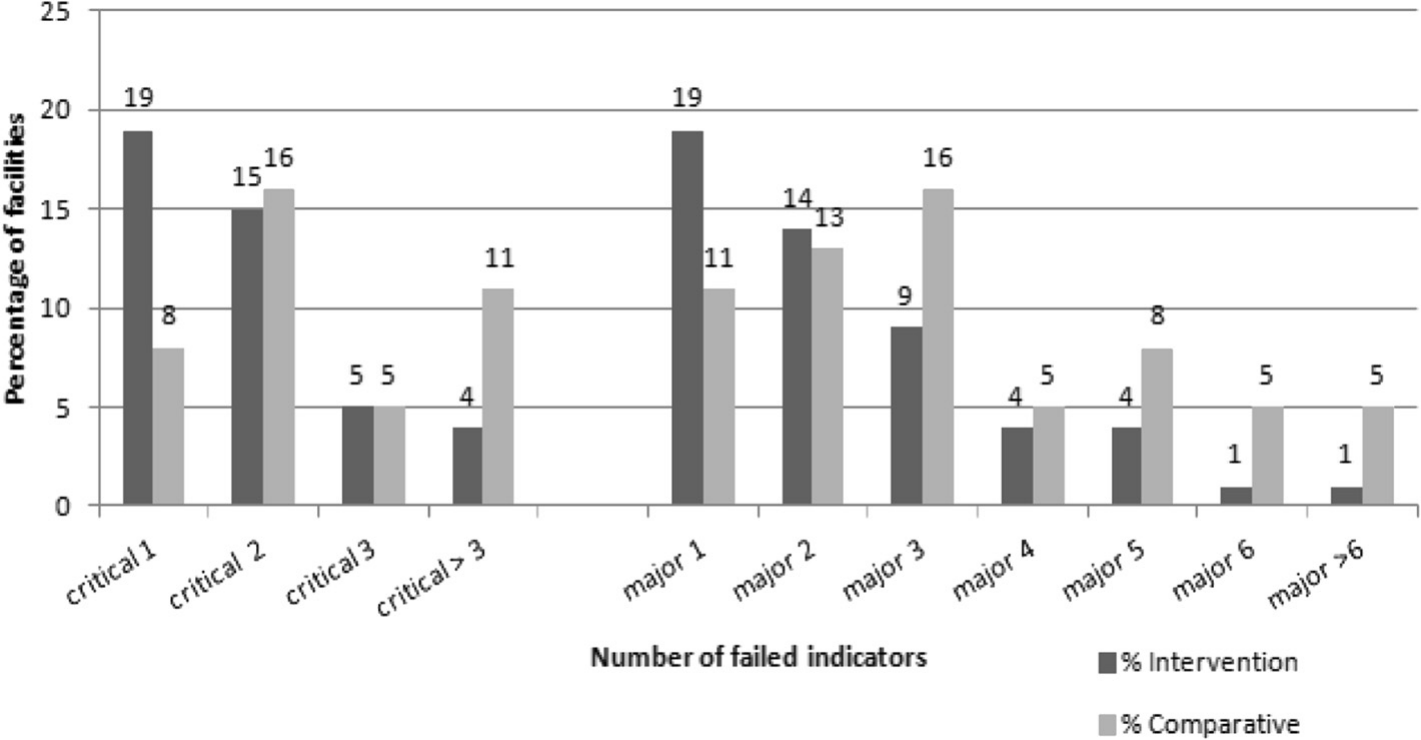
Comparison of intervention and comparative facilities by number of failed critical and major indicators

Facilities cannot be certified if they fail any critical indicator and over seven major indicators. Figure 1 displays the number of critical and major indicators failed in the intervention and comparative facilities including both certified and none certified. It shows that fewer uncertified intervention facilities than uncertified comparative facilities failed two or more critical and major indicators.

**Fig. 2:**
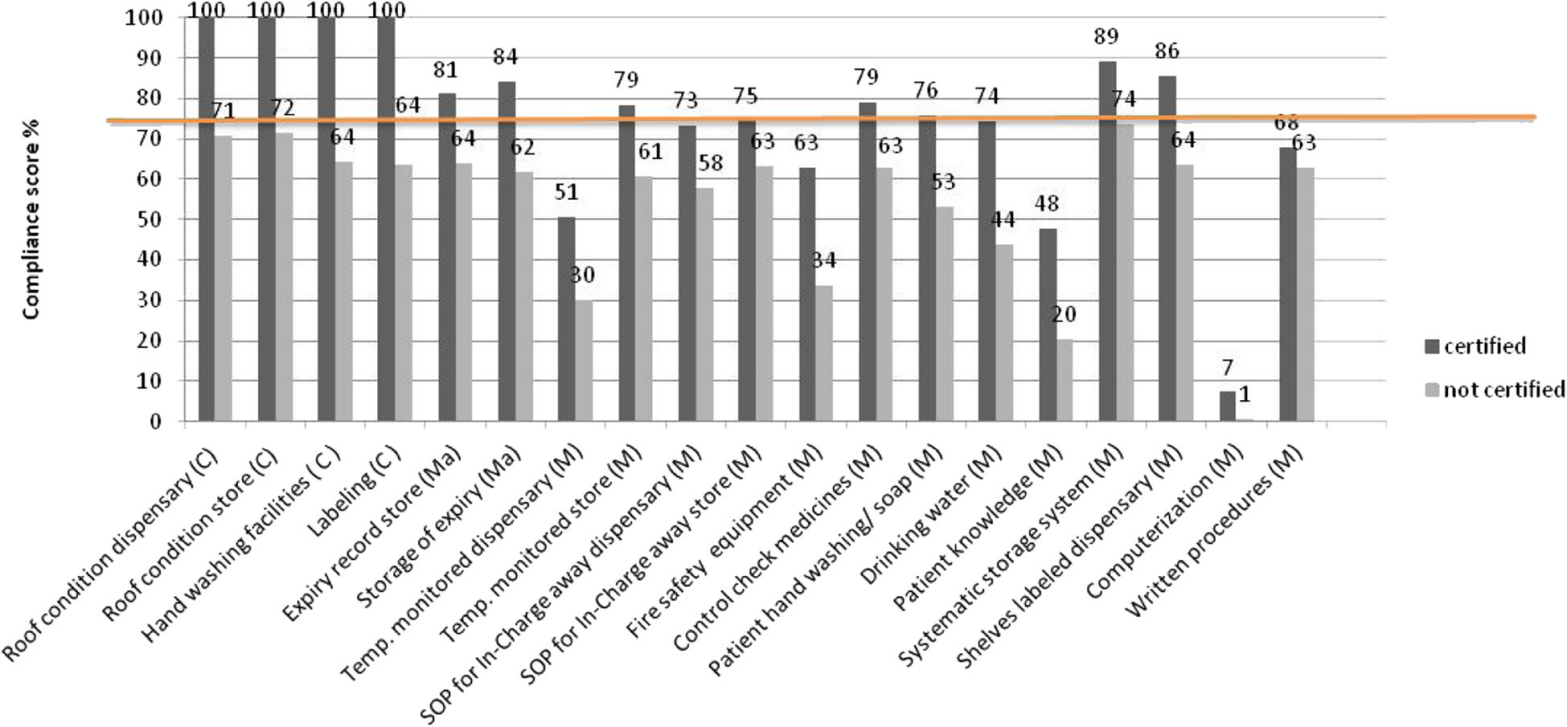
GPP indicators (C = Critical, Ma = Major and M = Minor indicators) identifying areas with challenges in adhering to GPP requirements identified by a indicator compliance score of below 75 % in SPARS-supported facilities, by certification status

To identify areas of none compliant GPP implementation in the SPARS supported facilities (intervention), we identified indicators failed by 25 % or more of the inspected facilities and classify these areas as challenging in regards to GPP implementation. Thus, if performance for a given indicator was less than 75 % facilities passing that indicator it fell within areas of challenge to GPP implementation. Challenging areas were identified for 19 (28 %) of the 67 indicators in the intervention group (Fig. 2). challenging areas identified by critical indicators covered: *availability of hand washing facilities, adherence to labeling requirements, and roof and ceiling conditions*. The challenging areas identified by major indicators were: *storage and recording of expired medicines*. The remaining challenging areas fell within the area measured by minor indicators that do not influence certification. Certified facilities had challenging GPP adherence areas identified by seven indicators (all minor), and uncertified facilities by 19 (four critical, two major, and 13 minor) indicators (Fig. 2).

The proportion of certified facilities passing each indicator was higher than that of uncertified facilities, and the differences were significant (*p* < adjusted 0.05) for 26 indicators (results not shown).

### Impact of SPARS intervention

To investigate the impact of SPARS on GPP adherence, we compared the number of failed GPP indicators as well as certification status between SPARS-supported and comparative facilities.

#### Effect of SPARS on compliance scores

**Fig. 3:**
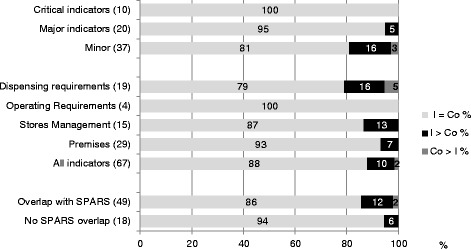
GPP indicator scores, by assessment area and by direction of significant differences between I (intervention - SPARS supported facilities) and Co (comparative facilities)

Figure 3 shows that the group of SPARS-supported facilities performed significantly better (*p* < adjusted 0.05) than the comparative group in 3 of the 4 assessment areas (dispensing requirements, stores management and premises) and for two categories of indicators (major, minor). Overall, intervention facilities outperformed significantly comparative facilities for 7 (10 %) indicators (I > Co), 59 (88 %) of the indicators (I = Co) did not differ, and comparative facilities scored higher for 1 (2 %) indicators (I < Co). SPARS-supported facilities outscored the comparative group on overlapping and none overlapping indicators (12 % vs. 2 % and 6 % vs. 0 % respectively).

An additional file present the compliance score for each indicator in the two arms [See Additional file 2].

**Fig. 4:**
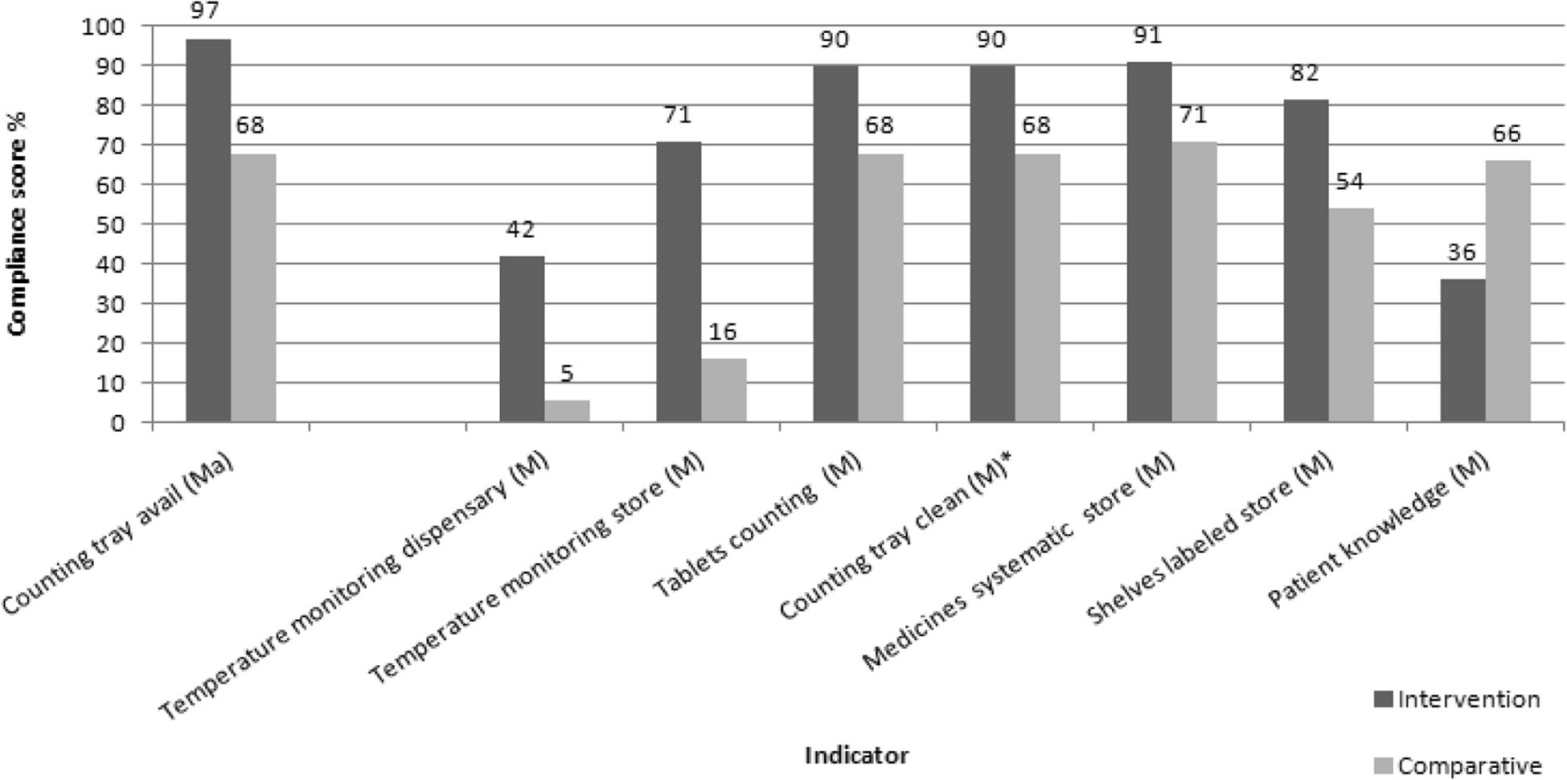
GPP indicators with significant differences in compliance score between intervention (SPARS supported) and comparative facilities. Indicators classified as Major (Ma) and Minor (M). *Identify indicators that are different (no overlap) from SPARS indicators

Figure 4 depicts the eight indicators (7 with I > Co and 1 with Co > I) with significantly different performance between intervention and comparative facilities. No significant differences were seen between the two arms for any of the critical indicators of which one indicator (labeling) is influenced through SPARS supervision. The intervention group scored higher for one *major* and *6 minor* indicators, and performed on average 30 % better than comparative facilities for these indicators (range 20 to 55 %). The comparative group scored higher for 1 minor indicator by 30 %.

#### Effect of SPARS on the number of failed indicators

Table 3 displays the average number of failed indicators by certification status comparing intervention and comparison facilities. A perfect score is zero indicating all GPP indicators passed. Comparative facilities failed more indicators than intervention facilities, 13 versus 9 indicators respectively (*p* = 0.017). Comparing facilities failing certification showed that SPARS supported facilities failed fewer indicators compared to the comparison facilities, 13 versus 18 (*p* = 0.003). On average, a certified intervention facility failed 6 indicators (out of 67 indicators), while a certified comparative facility failed 9 indicators. For certified facilities the difference between SPARS supported and comparative facilities were only significant for *certification with major comments*, with SPARS supported facilities failing 8 compared to 13 indicators (*p* = 0.026).

**Table 3:** GPP certification status: Average number of indicators failed in comparative and intervention facilities

	Overall (*n* = 455)			Intervention (n-417)			Comparative (*n* = 38)			
Certification status	n	Average No. of failed indicators (SD)	95 % Conf. Interval	n	Average No. of failed indicators (SD)	95 % Conf. Interval	n	Average No. of failed indicators (SD)	95 % Conf. Interval	*p*-value
Certification without comments	1	0					1	0		
Certification with minor comment	126	4.19 (2.6)	3.74–4.64	115	4.08 (2.6)	3.60–4.56	11	5.36 (1.8)	4.23–6.50	0.116
Certification with major comment	134	9.01 (5.1)	8.14–9.87	123	8.62 (4.8)	7.75–9.48	11	13.36 (5.7)	9.85–16.88	0.026
Certified^a^	261	6.65 (4.7)	6.07–7.22	238	6.42 (4.5)	5.84–7.00	23	8.96 (6.0)	6.35–11.56	0.062
Failed GPP certification	194	13.64 (6.0)	12.79–14.49	179	13.27 (5.8)	12.41–14.12	15	18.07 (6.8)	14.30–21.84	0.003
Total^a^	455	9.63 (6.3)	9.04–10.21	417	9.36 (6.1)	8.77–9.95	38	12.55 (7.7)	10.02–15.09	0.017

^a^ *t*-test with unequal variances; SD: standard deviation

## Discussion

Our study investigated adherence to GPP in the public sector and the effect of a national capacity building strategy SPARS on GPP implementation in Uganda, from data collected during the first NDA inspections carried out in the public sector.

Our study explored the adequacy of the redesigned NDA inspection tool for assessing GPP implementation in government facilities. The tool allowed collecting data that can be used as baseline in future interventions. It has formed the basis for developing strategies to improve adherence to GPP requirements at facility level and thereby increase GPP accreditation. In addition, using the same tool to inspect government, PNFP, and private sector pharmacies allows quantifying and comparing facility improvements and GPP accreditation status over time and across sectors.

Appropriate data quality was ensured by selecting experienced inspectors who were trained to use the electronic, standardized, and indicator-based inspection data collection form. Completeness of data was occasionally problematic, but the intervention sample was large enough to allow for exclusion of facilities with incomplete data. The excluded facilities were similar to the included in regards to level of care, ownership and region, suggesting that exclusion would not influence the results. Continued training of inspectors is recommended.

Experience with electronic data entry combined with the use of a central database was optimal in standardizing the assessment. Inspectors were able to enter data while on site and see the results instantaneously: this was critical to ensure data quality and completeness. Electronic data entry is a solution to the increased workload created by the increased number of inspections. Building additional features such as a spider graph or histogram of inspection data for each facility would undoubtedly help facilities visualize their GPP status in real time and track improvements for their staff [5, 7, 16].

Our results indicate that GPP are insufficiently implemented in the public sector both at government as well as PNFP sector facilities, a situation similar to that in Laos [8]. Overall, only 57 % of public facilities met required GPP standards and were certified to practice pharmacy. Our study suggests the need to complement SPARS interventions with inspections as a regulatory strategy to continually monitor facilities’ progress [7, 21, 22].

Hospitals tended to outperform lower level of care facilities, possibly because certification rules favor better equipped facilities: a facility obtains accreditation only if it passes all critical indicators and all but one critical indicator depend on building infrastructure. Ensuring good performance in this area requires financial investments to implement infrastructural improvements related to roofing, walls, water and sanitation, and spacing. Most other areas where GPP is found challenging can be addressed by behavioral and procedural changes. The relatively higher proportion of hospitals included in the comparative group (10.5 %) than in the intervention group (5 %) could have confounded these results as they are generally better resourced. However, the certification results and differences between groups did not change after excluding all hospital level data from the analysis (results not shown).

Similarly, one could have expected PNFP facilities to outperform Government facilities because of donor and patient funding sources. That however was not found, possibly because the majority of the PNFP lower level facilities are situated in most poor and rural settings with low self financing and cost recovery capacity.

The Northern Region outperformed the other regions with regards to GPP certification independent of exposure to SPARS. SPARS implementation was also better in this region, with no immediate explanation [17].

The stringent rule of certifying only facilities that pass all critical indicators may also explain the lack of difference in certification between the two groups, even though SPARS-supported facilities outperformed comparative facilities otherwise. The certified SPARS supported facilities failed an average of six indicators compared to nine indicators for comparative, and majority of them failed only one critical and one major indicator compared to comparative’s failing three or more critical and major indicators.

Another possible explanation for the lack of difference in certification is the size of the reference sample which was considerably smaller than the intervention group and for this reason probably not representative even though it was comparable in terms of level of care and type of facilities (government or PNFP). The small number of the comparative sample reduces the power of this study to find significant difference between intervention and comparative facilities. A comparative sample of 388 facilities would have been necessary to detect a difference of 10 % between the two arms with a power of 80 %.

Our study suggests the benefit of preparing facilities for accreditation through a GPP strengthening strategy, such as SPARS. SPARS-supported facilities performed better than comparative facilities, had higher overall GPP compliance score and outperformed the comparative facilities in passing major, and minor indicators. There was no difference in compliance score for critical indicators between the two arms. One explanation could be that critical indicators mainly assess structural conditions, such as wall, floors, roofs, water and sanitation for which supervision has limited influence. Further, of the indicators in which intervention facilities outperformed comparative facilities, all were indicators covered and regularly monitored by the SPARS intervention except for cleanliness of the counting tray that is not part of the SPARS indicators or supervision. Shelving was provided as part of SPARS, and most intervention facilities are now able to store medicines in a more systematic and organized manner, which underscores the considerable impact of improving infrastructure. Comparative facilities outperformed intervention facilities for one indicator covered by the SPARS intervention: patient knowledge, an indicator that much depends on availability of dispensing envelopes known to frequently be out of stock at lower level facilities.

SPARS and GPP focus areas overlap in 49 (73 %) of the indicators. These areas are stores management, premises management, and dispensing, with little overlap in operating requirements. These areas and indicators of overlap are correlated with the intervention group’s higher performance scores. However, intervention facilities only outperformed comparative facilities in 6 (12 %) of the 49 overlapping indicators. Most of the other 43 overlapping indicators were already well implemented before the study, explaining why 27 (63 %) of them scored above 85 % in both groups, decreasing the chance to observe any difference. Though SPARS indicators assess GPP implementation and identify deficiencies, resource allocation is management responsibility and SPARS strategy is limited to inexpensive corrective actions through in service training and behavior change. Moreover, there is still room for improvement after four SPARS visits.

The main limitation of the study is its cross sectional design and the related inability to demonstrate overtime improvement in SPARS-supported facilities compared to comparative. In addition, the GPP certification implemented as a national strategy made it not possible to have an equal sized comparative sample. All NDA inspectors have performed GPP inspections over the past many years using an almost similar tool and there is no reason to expect differences in the inspection outcome related to the time of inspection in the study period. One could expect inter-rater viability between the inspectors, however this was not investigated (Blick B, Nakabugo S, Seru M, Trap B. Evaluating inter-rater reliability of indicators to assess Ugandan health facility performance in medicines management. Unpublished). The exclusion of 38 facilities due to incomplete data for at least one critical indicator is unlikely to have influenced the results: these facilities did not differ from those included in the analysis with regards to level of care, region, ownership, and exposure to SPARS. Finally, the differences between SPARS and certification indicators created analytical challenges in testing the relationship between SPARS and GPP certification.

## Conclusion

Our study documents the need for establishing regular GPP inspections in the public sector as only 57 % of public sector facilities meet GPP certification criteria. Generally, GPP is insufficiently implemented in government and private not-for-profit facilities.

Our results suggest the value of preparing facilities for accreditation through a GPP-strengthening strategy. Specifically, SPARS-supported facilities had better overall GPP implementation across all categories of indicators (critical, major, and minor). Higher performance was most pronounced in GPP areas supported by SPARS supervision. A capacity building strategy, such as SPARS, combined with a regulatory intervention, such as GPP inspections and infrastructural development, are important instruments to improve GPP implementation and equity in Uganda.

## Additional files

Additional file 1: GPP inspection indicators with classification (critical, major, and minor) and overlap with SPARS indicators indicated with*, partly overlap **. (DOCX 145 kb) Additional file 2: Comparison of compliance score between intervention (I) and comparative (C) facilities, by domain and GPP indicator. I = C compliance scores are equal, I > C: Intervention scores significantly higher (*p* < 0.05adj.) and C > I: Comparison scored significantly (*p* < 0.05 adj.) higher. (PDF 210 kb)

## Abbreviations

C: critical
Co: comparative
DH: District Hospitals
GPP: Good Pharmacy Practices
HC: Health Centers
I: intervention
M: minor
Ma: major
MOH: Ministry of Health
NDA: National Drug Authority
OR: odds ratio
PNFP: private not-for-profit
SD: standard deviation
SPARS: Supervision, Performance Assessment, and Recognition Strategy
USAID: United States Agency for International Development
