# Supplementary material for: First regulatory inspections measuring adherence to Good Pharmacy Practices in the public sector in Uganda: a cross-sectional comparison of performance between supervised and unsupervised facilities
Source: J Pharm Policy Pract. 2016 May 4;9:18. doi: 10.1186/s40545-016-0068-4 (PMC4857441; doi:10.1186/s40545-016-0068-4)

Annex 1. GPP inspection indicators with classification (critical, major, and minor) and overlap with SPARS indicators indicated with*, partly overlap **.


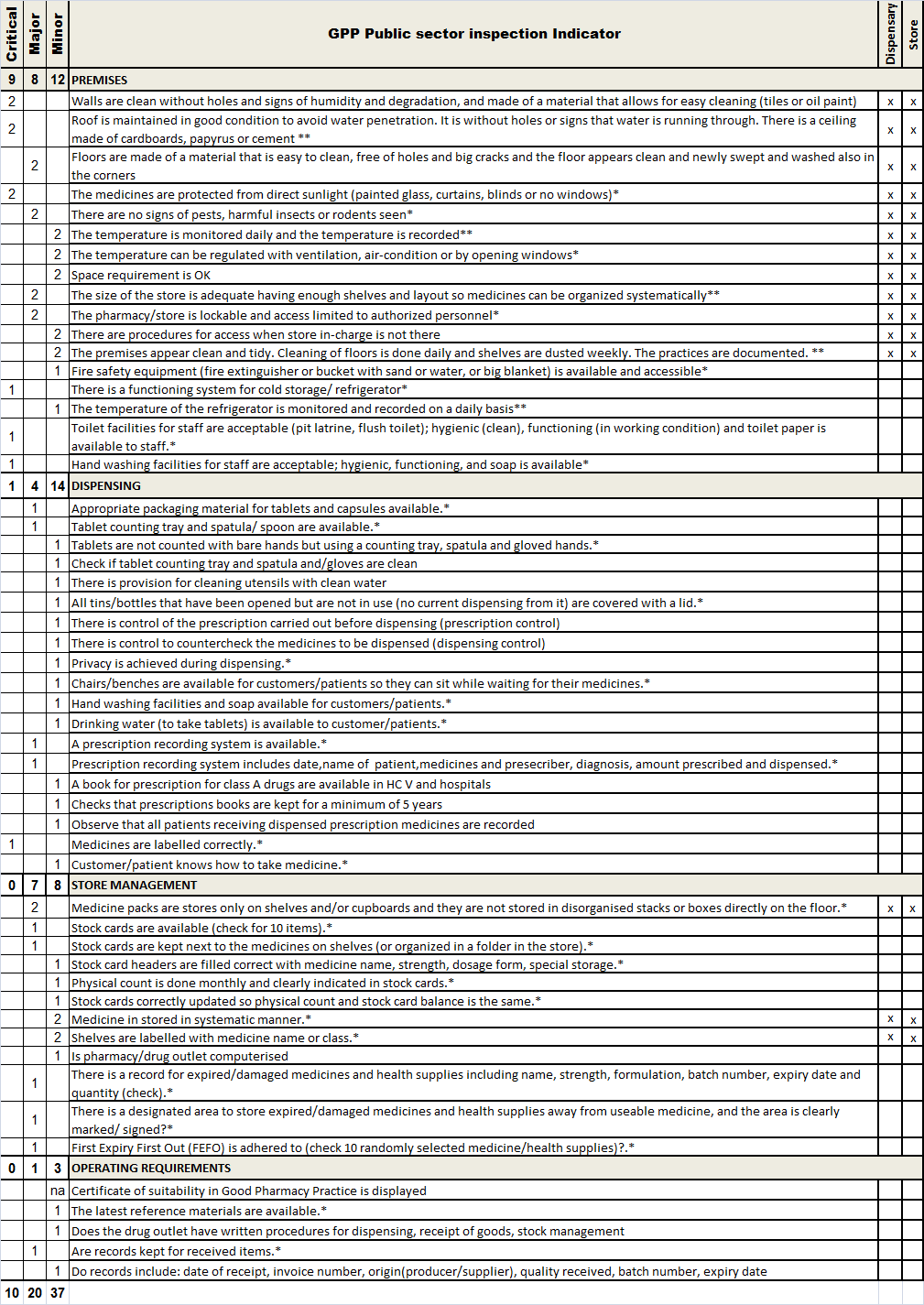

Supplement: Additional file 1: — GPP inspection indicators with classification (critical, major, and minor) and overlap with SPARS indicators indicated with*, partly overlap **. (DOCX 145 kb) [file 40545_2016_68_MOESM1_ESM.docx]
