# Supplementary material for: First regulatory inspections measuring adherence to Good Pharmacy Practices in the public sector in Uganda: a cross-sectional comparison of performance between supervised and unsupervised facilities
Source: J Pharm Policy Pract. 2016 May 4;9:18. doi: 10.1186/s40545-016-0068-4 (PMC4857441; doi:10.1186/s40545-016-0068-4)
Supplement: Additional file 2: — Comparison of compliance score between intervention (I) and comparative (C) facilities, by domain and GPP indicator. I = C compliance scores are equal, I > C: Intervention scores significantly higher (p < 0.05adj.) and C > I: Comparison scored significantly (p < 0.05 adj.) higher. (PDF 210 kb) [file 40545_2016_68_MOESM2_ESM.pdf]

GPP Public sector inspection Indicator					I=C	I>C	I<C
**PREMISES**		**Co**	**I**	**p-value***	**59**	**7**	**1**
Walls are clean without holes and signs of humidity and degradation, and made of a material that allows for easy cleaning (tiles or oil paint)	Dispensary	89.5	96.6	0.0539893	1		
	Store	89.5	97.1	0.0362959	1		
Roof is maintained in good condition to avoid water penetration. It is without holes or signs that water is running through. There is a ceiling made of cardboards, papyrus or cement	Dispensary	73.7	87.5	0.0172321	1		
	Store	68.4	87.8	0.0009463	1		
Floors are made of a material that is easy to clean, free of holes and big cracks and the floor appears clean and newly swept and washed also in the corners	Dispensary	97.4	97.4	1.0000000	1		
	Store	89.5	97.8	0.0176755	1		
The medicines are protected from direct sunlight (painted glass, curtains, blinds or no windows)	Dispensary	97.4	95.7	1.0000000	1		
	Store	100.0	95.7	0.3847815	1		
There are no signs of pests, harmful insects or rodents seen	Dispensary	86.8	92.2	0.2448856	1		
	Store	84.2	90.5	0.2159402	1		
The temperature is monitored daily and the temperature is recorded	Dispensary	5.3	41.8	**0.0000001**		1	
	Store	15.8	70.8	**0.0000000**		1	
The temperature can be regulated with ventilation, air-condition or by opening windows	Dispensary	92.1	96.2	0.2022120	1		
	Store	94.7	95.3	0.7008249	1		
Space requirement is OK	Dispensary	94.7	91.9	0.7559942	1		
	Store	79.0	84.8	0.3476461	1		
The size of the store is adequate having enough shelves and layout so medicines can be organized systematically	Dispensary	94.7	91.5	0.7567958	1		
	Store	84.2	93	0.0539539	1		
The pharmacy/store is lockable and access limited to authorized personnel	Dispensary	92.1	94.2	0.4843495	1		
	Store	97.4	98.3	0.5132641	1		
There are procedures for access when store in-charge is not there	Dispensary	81.6	66.8	0.0623921	1		
	Store	76.3	71	0.4911890	1		
The premises appear clean and tidy. Cleaning of floors is done daily and shelves are dusted weekly. The practices are documented	Dispensary	97.4	89.9	0.2387767	1		
	Store	94.7	89.1	0.4062843	1		
Fire safety equipment (fire extinguisher or bucket with sand or water, or big blanket) is available and accessible		31.6	50.4	0.0266514	1		
There is a functioning system for cold storage/ refrigerator		100.0	97.2	0.6057635	1		
The temperature of the refrigerator is monitored and recorded on a daily basis		96.4	96.8	1.0000000	1		
Toilet facilities for staff are acceptable (pit latrine, flush toilet); hygienic (clean), functioning (in working condition)		89.5	90.9	0.7685676	1		
Hand washing facilities for staff are acceptable; hygienic, functioning, and soap is available		94.7	84.6	0.1441596	1		
**DISPENSING**							
Appropriate packaging material for tablets and capsules available.		92.1	99.3	0.0090447	1		
Tablet counting tray and spatula/ spoon are available		68.4	96.6	**0.0000000**		1	
Tablets are not counted with bare hands but using a counting tray, spatula and gloved hands		68.4	89.6	**0.0001266**		1	
Check if tablet counting tray and spatula and/gloves are clean		68.4	89.8	**0.0001002**		1	
There is provision for cleaning utensils with clean water		86.8	85.3	0.8003316	1		
All tins/bottles that have been opened but are not in use (no current dispensing from it) are covered with a lid		92.1	95.2	0.4302516	1		
There is control of the prescription carried out before dispensing (prescription control)		97.4	79.3	0.0042001	1		
There is control to countercheck the medicines to be dispensed (dispensing control)		94.7	72.2	0.0015208	1		
Privacy is achieved during dispensing		92.1	88.7	0.7857912	1		
Chairs/benches are available for customers/patients so they can sit while waiting for their medicines		92.1	96.4	0.1840003	1		
Hand washing facilities and soap available for customers/patients		79.0	66.1	0.1065627	1		
Drinking water (to take tablets) is available to customer/patients		50.0	61.4	0.1687879	1		
A prescription recording system is available		97.4	99.5	0.2320727	1		
Prescription recording system includes date, name of patient, medicines and prescriber, diagnosis, amount prescribed		97.4	96.6	1.0000000	1		
A book for prescription for class A drugs are available in HC V and hospitals		94.7	85.0	0.2211745	1		
Checks that prescriptions books are kept for a minimum of 5 years		97.4	98.7	0.4233129	1		
Observe that all patients receiving dispensed prescription medicines are recorded		92.1	93.5	0.7301176	1		
Medicines are labelled correctly		97.4	84.4	0.0283782	1		
Customer/patient knows how to take medicine		65.8	36.1	**0.0003175**			1
**STORE MANAGEMENT**							
Medicine packs are stores only on shelves and/or cupboards and they are not stored in disorganised stacks or boxes directly on the floor	Dispensary	94.7	93.1	1.0000000	1		
	Store	89.5	93.4	0.3197015	1		
Stock cards are available (check for 10 items).		92.1	99	0.0149158	1		
Stock cards are kept next to the medicines on shelves (or organized in a folder in the store).		92.1	98.6	0.0318052	1		
Stock card headers are filled correct with medicine name, strength, dosage form, special storage		78.9	91.8	0.0085429	1		
Physical count is done monthly and clearly indicated in stock cards		81.6	90.3	0.0904606	1		
Stock cards correctly updated so physical count and stock card balance is the same		89.5	92.4	0.5231189	1		
Medicine in stored in systematic manner	Dispensary	71.0	91.2	0.0845904	1		
	Store	71.0	91.2	**0.0001114**		1	
Shelves are labelled with medicine name or class	Dispensary	55.3	75.8	0.0059890	1		
	Store	52.6	87.4	**0.0000000**		1	
Is pharmacy/drug outlet computerised		15.8	4.4	0.0028316	1		
There is a record for expired/damaged medicines and health supplies including name, strength, formulation, batch		73.7	73.8	0.9878104	1		
There is a designated area to store expired/damaged medicines and health supplies away from useable medicine, and		68.4	74.5	0.4170590	1		
First Expiry First Out (FEFO) is adhered to (check 10 randomly selected medicine/health supplies)?		94.7	93.1	1.0000000	1		
**OPERATING REQUIREMENTS**							
Certificate of suitability in Good Pharmacy Practice is displayed					na		
The latest reference materials are available		68.4	81.6	0.0488476	1		
Does the drug outlet have written procedures for dispensing, receipt of goods, stock management		50.0	66.0	0.0493951	1		
Are records kept for received items		92.1	98.0	0.0590694	1		
Do records include: date of receipt, invoice number, origin(producer/supplier), quality received, batch number, expiry		100.0	94.2	0.2474476	1		
* p values are compared to the adjusted for critical values. Only bolded p valus are after adjustment significant					59	7	1
